# Atrial fibrillation and oral anticoagulation in older people with frailty: a nationwide primary care electronic health records cohort study

**DOI:** 10.1093/ageing/afaa265

**Published:** 2020-12-16

**Authors:** Chris Wilkinson, Andrew Clegg, Oliver Todd, Kenneth Rockwood, Mohammad E Yadegarfar, Chris P Gale, Marlous Hall

**Affiliations:** Leeds Institute of Cardiovascular and Metabolic Medicine, University of Leeds, Leeds, UK; Population Health Sciences Institute, Faculty of Medical Sciences, Newcastle University, Newcastle upon Tyne, UK; Academic Unit for Ageing and Stroke Research, Leeds Institute of Health Sciences, University of Leeds, Leeds, UK; Bradford Institute for Health Research, Bradford Teaching Hospitals NHS Foundation Trust, Bradford, UK; Academic Unit for Ageing and Stroke Research, Leeds Institute of Health Sciences, University of Leeds, Leeds, UK; Bradford Institute for Health Research, Bradford Teaching Hospitals NHS Foundation Trust, Bradford, UK; Geriatric Medicine, Dalhousie University, Halifax, Nova Scotia, Canada; Leeds Institute of Cardiovascular and Metabolic Medicine, University of Leeds, Leeds, UK; Leeds Institute for Data Analytics, University of Leeds, Leeds, UK; Leeds Institute of Cardiovascular and Metabolic Medicine, University of Leeds, Leeds, UK; Leeds Institute for Data Analytics, University of Leeds, Leeds, UK; Department of Cardiology, Leeds Teaching Hospitals NHS Trust, Leeds, UK; Leeds Institute of Cardiovascular and Metabolic Medicine, University of Leeds, Leeds, UK; Leeds Institute for Data Analytics, University of Leeds, Leeds, UK

**Keywords:** anticoagulation, atrial fibrillation, frailty, older people, stroke

## Abstract

**Background:**

Atrial fibrillation (AF) is common in older people and is associated with increased stroke risk that may be reduced by oral anticoagulation (OAC). Frailty also increases with increasing age, yet the extent of OAC prescription in older people according to extent of frailty in people with AF is insufficiently described.

**Methods:**

An electronic health records study of 536,955 patients aged ≥65 years from ResearchOne in England (384 General Practices), over 15.4 months, last follow-up 11th April 2017. OAC prescription for AF with CHA_2_DS_2_-Vasc ≥2, adjusted (demographic and treatments) risk of all-cause mortality, and subsequent cerebrovascular disease, bleeding and falls were estimated by electronic frailty index (eFI) category of fit, mild, moderate and severe frailty.

**Results:**

AF prevalence and mean CHA_2_DS_2_-Vasc for those with AF increased with increasing eFI category (fit 2.9%, 2.2; mild 11.2%, 3.2; moderate 22.2%, 4.0; and severe 31.5%, 5.0). For AF with CHA_2_DS_2_-Vasc ≥2, OAC prescription was higher for mild (53.2%), moderate (55.6%) and severe (53.4%) eFI categories than fit (41.7%). In those with AF and eligible for OAC, frailty was associated with increased risk of death (HR for severe frailty compared with fit 4.09, 95% confidence interval 3.43–4.89), gastrointestinal bleeding (2.17, 1.45–3.25), falls (8.03, 4.60–14.03) and, among women, stroke (3.63, 1.10–12.02).

**Conclusion:**

Among older people in England, AF and stroke risk increased with increasing degree of frailty; however, OAC prescription approximated 50%. Given competing demands of mortality, morbidity and stroke prevention, greater attention to stratified stroke prevention is needed for this group of the population.

## Key points

- The prevalence of atrial fibrillation (AF) in people aged 65 years or older in England in 2015 was 11.4% (*n* = 61,177).
- Frailty was more common among people with AF (89.5%, *n* = 54,734) compared with those without AF (55.4%, *n* = 263,356).
- Overall, oral anticoagulation (OAC) was prescribed in 53.1% (*n* = 30,916) of patients with AF and a CHA₂DS₂-VASc score of ≥$2$. OAC was more commonly prescribed in patients with frailty (adjusted odds ratio compared with fit, mild: 1.84 [95% confidence intervals 1.72–1.96]; moderate 2.34 [1.18–2.50] and severe 2.51 [2.33–2.71]).
- AF was associated with a 59% increased risk of mortality compared with patients without AF, regardless of the extent of their frailty.
- After accounting for prescription of OAC and patient and clinical demographics, frailty was associated with an increased risk of mortality, gastrointestinal bleeding and falls in patients with AF.

## Introduction

At least 10 million people in Europe have a diagnosis of atrial fibrillation (AF), which is associated with increased risk of stroke and mortality [1, 2]. In particular, AF is common amongst older people, affecting 7.2% of people aged over 65 years, and 15.1% of those aged between 85 and 89 years [3, 4]. Given its association with age, many people with AF also live with frailty—a condition in which there is a decline in biological reserves and deterioration in physiological mechanisms that render people vulnerable to a range of adverse outcomes [5,6]. Although appropriate prescription of oral anticoagulation (OAC) reduces stroke risk by ~64% and is associated with a mortality advantage, there may be prescribing inertia for OAC in those with frailty due to fears of iatrogenic harm [7–9].

Frailty is often more useful than chronological age in guiding individualised treatment of older people with cardiovascular disease in the context of advancing multi-morbidity and polypharmacy [10,11]. However, despite an increasing prevalence of both AF and frailty, comprehensive community-based data are lacking to quantify the disease burden, OAC prescription rates and clinical outcomes for older people with AF and frailty [12]. This work is particularly important given recent findings that the association between frailty and increased cardiovascular events and mortality appears to be independent of traditional cardiovascular risk factors [13]. Frailty may be identified using electronic health records (EHR) in primary care using an electronic frailty index (eFI) [14], based upon the cumulative deficit model [15]. The eFI has robust predictive validity for outcomes of mortality, hospitalisation and nursing home admission, and has convergent validity against established frailty instruments [16]. In this study, we describe the prevalence of both frailty and AF in patients aged 65 years or older; evaluate OAC prescription rates; and analyse clinical outcomes in patients with AF, according to the degree of frailty.

## Methods

### Data and participants

This population-based cohort study used data from the ResearchOne primary care database, which includes de-identified clinical and administrative data derived from the EHRs of patients registered at a general practice in England using the SystmOne clinical system [17]. As of 2016, there were 20.2 million patients registered in SystmOne, representing 35% of patients in England [18]. The analytical cohort included 50.5 million ResearchOne records dating back as far as 1 January 1986 for 536,955 patients aged 65 years or older by 31 December 2015. For each individual, GP records were followed up until death, moving from a ResearchOne practice, or the censoring date, 11th April 2017.

The AF group constituted those with a diagnosis of current or resolved AF (paroxysmal, persistent and permanent) or atrial flutter recorded in their primary care record on or at any time before 31st December 2015. Patients with a record of AF, but no date of diagnosis were excluded (Supplementary Figure 1). AF was defined according to a comprehensive list of clinical terms version-3 (CTV-3) codes (data supplement). Frailty was estimated using the eFI, in which the proportion of deficits (symptoms and signs, abnormal laboratory values, disability or disease state) from 36 possible deficits was calculated, categorised into fit (0–0.12), mild (>0.12–0.24), moderate (>0.24–0.36) or severe (>0.36) frailty [14]. With the exception of polypharmacy (≥5 prescriptions in preceding 12 months), deficits were identified from every EHR preceding 31st December 2015 using CTV-3 codes defined in the eFI [14].

The primary outcomes were all-cause mortality and first stroke (ischaemic or unspecified). Secondary outcomes were first gastrointestinal or intracranial bleed, fall and TIA (CTV-3 codes in the data supplement). Date of death from any cause (aggregated at source to month and year) was ascertained from the GP record and provided as part of the anonymised patient-level dataset. Stroke risk in patients with AF was calculated using the CHA₂DS₂-VASc score [19]. Scores range from zero to nine (higher scores indicating greater stroke risk). Participants with scores of two or more were considered eligible for OAC, in accordance with guidelines [20,21].

### Statistical analyses

Baseline characteristics including patient demographics (age, sex, postcode level indices of multiple deprivation (IMD) rank and residence in a nursing home) and medical history (cancer, chronic kidney disease, diabetes, heart failure, hypertension, hyperthyroidism, ischaemic heart disease, myocardial infarction, pulmonary embolism, stroke [ischaemic or unspecified], falls, valvular heart disease; and history of smoking [never vs. ever]) were described for patients with and without AF. Numbers and percentages were presented for categorical variables; means with standard deviations (SD) or medians and interquartile range (IQR) for normally and non-normally distributed continuous variables respectively.

A point-prevalence of AF with 95% confidence intervals (CI) on 31 December 2015, and the proportion of those with AF who were prescribed OAC (direct oral anticoagulant [DOAC] or warfarin) of those eligible to receive treatment were calculated. To determine the association between frailty and OAC prescription, logistic regression models with binary treatment outcome and categories of frailty were fitted with adjustment for factors that may affect the risk of bleeding or interact with anticoagulants: age; medical history of cancer, varices, bleeding (gastrointestinal or intracranial), co-prescription of an antiplatelet medication, and prescription of steroid, non-steroidal anti-inflammatory drug, macrolide antibiotic or proton-pump-inhibitor in the previous year [22]. To account for practice level variation, models included adjustment for GP practice identifiers (ID). An unadjusted scatter plot showing the proportion of patients prescribed OAC for a given eFI was generated (Supplementary Figure 2).

**Figure 1:**
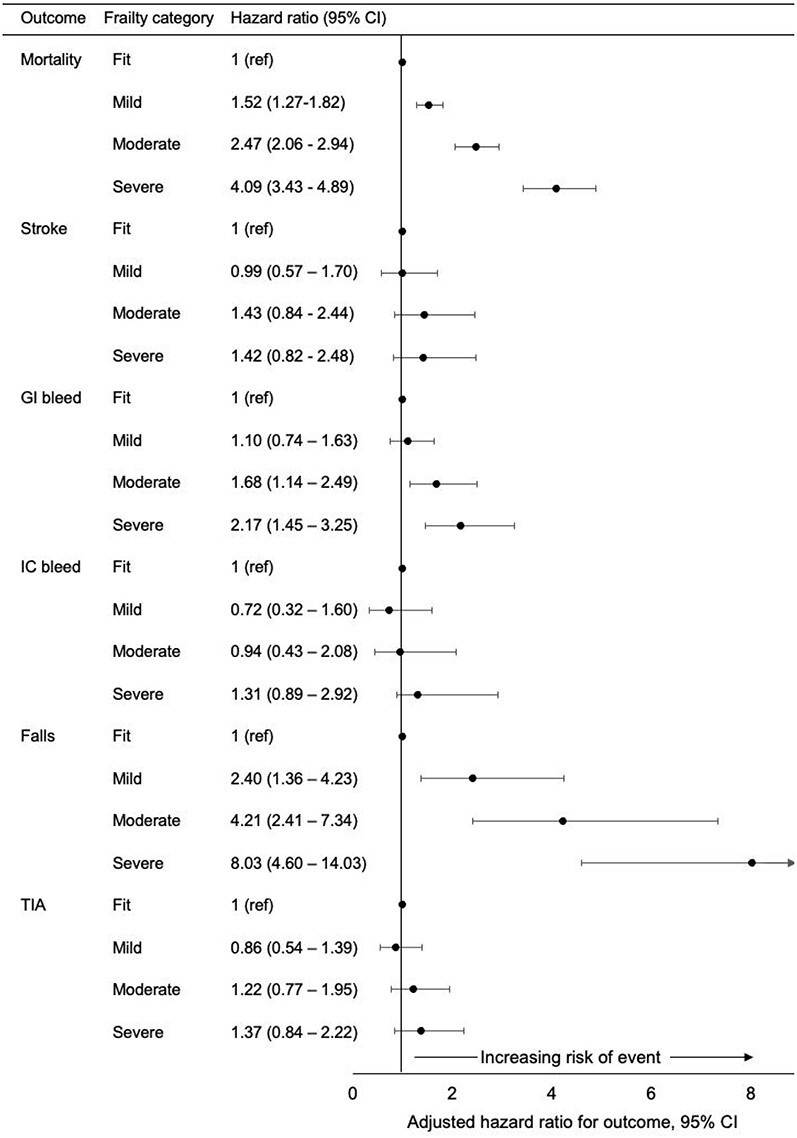
Adjusted association between frailty category and clinical outcomes in patients with atrial fibrillation and eligible for oral anticoagulation. Adjusted for age, sex, smoking, indices of multiple deprivation, general practice identifier, oral anticoagulation and antiplatelet prescription. Mortality was modelled as a competing risk for non-death outcomes. **Abbreviations** GI: gastrointestinal; IC: intracranial; TIA: transient ischaemic attack. Unadjusted estimates (including for participants without AF) are reported in Supplementary Tables 3–5.

Incidence rates of the first occurrence of each clinical outcome were reported for individuals with and without AF by frailty category and expressed per 1,000 person-years (/1,000 pys). A series of Cox proportional hazards models were fitted to determine the risk of mortality for patients with and without AF (adjusted for age, sex, smoking, IMD and GP practice), and for each outcome, stratified by frailty category, amongst people with AF, and limited to those with AF and eligible for OAC. Proportionality of hazards was confirmed using a log–log survival plot, and the risk of each outcome was estimated in the whole cohort after adjusting for age, sex, smoking status and IMD at time of study entry and practice ID. In patients with AF and CHA_2_DS_2_Vasc ≥2 estimates were further adjusted for prescription of antiplatelet and OAC. In this subgroup, Fine and Gray competing risk models were implemented treating death as a competing risk for non-fatal outcomes (presented in Figure 1) [23], and because of increasing evidence for sex inequalities in frailty-related outcomes, we stratified the primary analyses by sex [24]. Missing data for baseline demographics were minimal (IMD missing in 6%), and all other data were collected on a positive recording basis (whereby the absence of a recorded diagnosis is treated as the absence of that event). Therefore, no formal missing data strategy was employed. Analyses were undertaken using Stata MP 14.0 (StataCorp LP, USA) with statistical significance determined at *P* < 0.05.

The study proposal was approved by the ResearchOne Project Committee (REC 11/NE/0184). As we made secondary use of pseudonymised patient level data collected in the course of normal care, no further NHS research ethics committee approval was required.

## Results

The analytic cohort comprised 536,955 patients (54.2% women, median age 73.8 years) from 384 practices in England (Table 1). Data were missing for IMD in 32,336 (6.0%), with no missing sex or age data. The prevalence of AF at study entry was 11.39% (95% CI 11.31–11.48%, *n* = 61,177), and was higher with increased frailty (supplementary Table 1). Of those with AF, 89.5% (*n* = 54,734) had mild, moderate or severe frailty, compared with 55.4% (*n* = 263,356) of individuals without AF. There were 671,135 person-years of follow-up.

**Table 1:** Characteristics of patients by atrial fibrillation status at study entry

	All *n* = 536,955	No AF *n* = 475,778	AF *n* = 61,177
Age. Median (IQR)	73.8 (69.0–80.5)	73.1 (68.8–79.6)	79.7 (73.3–85.5)
Women *n* (%)	290,764 (54.2)	262,777 (55.2)	27,987 (45.8)
IMD *n* (%)			
Most deprived quintile	65,337 (13.0)	57,898 (13.0)	7,439 (12.9)
Least deprived quintile	122,726 (24.3)	109,281 (24.5)	13,445 (23.3)
Number of eFI deficits, median (IQR)	5 (3–8)	5 (3–8)	9 (6–12)
**Frailty category** *n* (%)			
Fit	218,865 (40.8)	212,422 (44.7)	6,443 (10.5)
Mild	181,986 (33.9)	161,634 (34.0)	20,352 (33.3)
Moderate	91,411 (17.0)	71,096 (14.9)	20,315 (33.2)
Severe	44,693 (8.3)	30,626 (6.4)	14,067 (23.0)
**Past medical history**			
Cancer	71,418 (13.3)	61,193 (12.9)	10,225 (16.7)
Chronic kidney disease	102,529 (19.1)	82,204 (17.3)	20,325 (33.2)
Diabetes	92,146 (17.2)	77,915 (16.4)	14,231 (23.3)
Heart failure	25,553 (4.8)	13,103 (2.8)	12,450 (20.4)
Hypertension	283,517 (52.8)	242,177 (51.0)	41,340 (67.6)
Hyperthyroid	10,875 (2.0)	8,873 (1.9)	2,002 (3.3)
Ischaemic heart disease	84,237 (15.7)	64,651 (13.6)	19,586 (32.0)
Myocardial infarction	32,802 (6.1)	25,383 (5.3)	7,419 (12.1)
Pulmonary embolism	9,597 (1.8)	7,718 (1.6)	1,879 (3.1)
Stroke	25,412 (4.7)	18,173 (3.8)	7,239 (11.8)
Infarct	10,593 (2.0)	7,414 (1.6)	3,179 (5.2)
Unspecified	17,982 (3.4)	12,939 (2.7)	5,043 (8.2)
Falls	56,407 (12.2)	53,649 (11.3)	11,758 (19.2)
Valvular heart disease	23,003 (4.3)	14,263 (3.0)	8,740 (14.3)
History of smoking	378,646 (70.7)	333,270 (70.3)	45,376 (74.3)

**Abbreviation**: *n*, number.

### Oral anticoagulation

Of patients with AF, 95.1% (*n* = 58,204) had a CHA_2_DS_2_-Vasc score of ≥2 and were considered eligible for OAC prescription, which was prescribed in 30,916 (53.1%) (Supplementary Table 2). Of those prescribed OAC, 23.7% (*n* = 7,329) were prescribed a DOAC. The mean CHA_2_DS_2_-Vasc score in AF patients eligible for OAC was higher with increased frailty category (fit 2.2 [SD 0.98]; mild 3.2 [1.2]; moderate 4.0 [1.3] and severe 5.0 [1.4]). Frailty category was positively associated with prescription of OAC (adjusted odds ratio compared with fit, mild: 1.84 [95% CI 1.72–1.96]; moderate 2.34 [1.18–2.50] and severe 2.51 [2.33–2.71], Supplementary Figure 2).

### Clinical outcomes

#### All-cause mortality

All-cause mortality rates were higher in patients with AF compared with patients without AF (Table 2, adjusted HR 1.59 [95% CI 1.55–1.64]). Frailty was associated with increased mortality for patients with AF and a CHA_2_DS_2_-Vasc score of ≥2 (Figure 1). The magnitude of association was larger in women (adjusted HR compared with fit, mild: 1.80 [95% CI 1.33–2.42]; moderate: 2.64 [1.97–3.55]; severe: 4.36 [3.25–5.85]) than in men (mild: 1.36 [1.08–1.70]; moderate: 2.36 [1.89–2.95]; severe: 3.97 [3.17–4.96]).

**Table 2:** Rates of clinical outcomes by AF status

	**No AF**, *n* = 475,778		**AF**, *n* = 61,177		*P* value
	Events (*n*)	Rate (95% CI)	Events (*n*)	Rate (95% CI)	
Death	18,111	30.34 (29.90, 30.79)	6,143	82.75 (80.70, 84.84)	<0.001
Stroke	1,293	2.17 (2.05, 2.29)	398	5.38 (4.87, 5.93)	<0.001
Infarct	696	1.17 (1.08, 1.26)	213	2.87 (2.51, 3.29)	<0.001
Unspecified	705	1.18 (1.10, 1.27)	211	2.85 (2.49, 3.26)	<0.001
Gastrointestinal bleed	2,653	4.46 (4.29, 4.63)	583	7.90 (7.29, 8.57)	<0.001
Intracranial bleed	493	0.83 (0.76, 0.90)	136	1.83 (1.55, 2.17)	<0.001
Falls	4.803	8.07 (7.84, 8.30)	1,421	19.23 (18.26, 20.26)	<0.001
Transient ischaemic attack	1,992	3.34 (3.20, 3.49)	372	5.03 (4.54, 5.57)	<0.001

Rates per 1,000 person years, with 95% CI.

#### Stroke

The risk of stroke was 67% higher in participants with AF compared with those without (adjusted HR 1.67, 95% CI 1.48–1.88). After adjustment, and accounting for competing risk of mortality, frailty was not associated with increased stroke risk for patients with AF and eligible for OAC (Figure 1). When the competing risks analysis was stratified by sex, women with moderate or severe frailty had an increased risk of stroke (adjusted HR comparing with fit, mild: 2.31 [0.70–7.57], moderate: 3.94 [1.22–12.75] and severe 3.63 [1.10–12.02]), but this was not the case in men (adjusted HR comparing with fit, mild: 0.69 [0.37–1.28], moderate: 0.83 [0.45–1.54] and severe: 0.85 [0.45–1.63]).

#### Gastrointestinal and intracranial bleeding

The rates of gastrointestinal and intracranial bleeding were higher in patients with AF than in those AF (Table 2). In patients with AF and eligible for OAC the moderate and severe frailty categories were at increased risk of GI bleed compared with the fit group, but there was no association between frailty category and IC bleeding events (Figure 1).

#### Falls

The rate of falls was higher in patients with AF than in those without (Table 2). In patients with AF and eligible for OAC, frailty was significantly associated with an increased risk of falls. Participants in the severe frailty category were over eight times more likely to fall than the fit group (adjusted HR 8.04 [4.60–14.03], Figure 1).

#### TIA

The rate of TIA was higher in patients with AF than those without (Table 2). In patients with AF and eligible for OAC, the adjusted HR did not vary significantly by frailty category (Figure 1).

## Discussion

In this community-based study of 536,995 older people, one in nine had a diagnosis of AF—of whom almost 90% had concomitant frailty. Patients with AF had higher rates of all-cause mortality, stroke, bleeding, TIA and falls than those without AF. Although overall OAC was prescribed in just half of eligible patients with AF, OAC prescription was more common in patients with frailty. After accounting for demographic profiles and treatment provision, patients with AF and a CHA_2_DS_2_-Vasc score of ≥2 had an elevated risk of mortality, GI bleeding, and falls with increasing frailty levels. Women, but not men, had an increased stroke risk associated with increased levels of frailty.

Stroke is a devastating condition—but in patients with AF it is largely preventable through appropriate use of OAC. Yet, prescription rates of OAC are low in eligible patients, as in other studies [25–29]. In general, stroke disease is significantly more common in older people than is intracranial bleeding, even in the presence of OAC [30, 31]. However, in this study, people with frailty more commonly had falls. Although guidance suggests that OAC should not be withheld solely because a person is at risk of falls, clinicians are fearful of the risk of harm from head injury in those that fall [21, 32, 33]. Other bleeding complications may be poorly tolerated in people with frailty—who have a lower physiological reserve to mount a full recovery [5, 34]. These must be set against a higher absolute risk of stroke, and therefore more needs to be done to understand and overcome reasons for non-prescription of OAC [35].

Interestingly, in this study we showed a positive association between frailty and prescription rates, although prescribing rates plateaued and dropped with more severe frailty. Previous small studies have shown conflicting findings—with one reporting a positive [36] and one a negative association between OAC prescription and phenotype-defined frailty status [37]. The present study has the advantages of a large, representative community-based dataset and a validated measure to ascertain frailty status [16].

Despite a growing number of patients with AF and frailty, data are lacking regarding the relative safety and efficacy of OAC in this population [12]. Observational evidence suggests the benefits of OAC may be similar for patients with and without frailty [37]. Although people with moderate–severe frailty are not well represented in randomised clinical trials of the efficacy and safety of DOACs, recent post-hoc analyses have shown that rates of stroke or systemic embolism, death, and major bleeding are increased with increased levels of frailty [38,39]. However, the efficacy and safety of apixaban compared with warfarin was not modified by multi-morbidity status [38], and a reduction in bleeding was shown with edoxaban compared with warfarin overall, but not significantly so in those with more advanced frailty (who made up a small proportion of the overall trial population) [39].

We have shown that frailty is common among patients with AF and is associated with a poor prognosis. Our finding that the risk of stroke is higher for women with increasing frailty, but not men, is of interest. Women tend to develop stroke disease at older ages than men [40], and tend to live longer with a greater degree of co-morbidity and frailty [24,41]. That many of the age-related deficits are risk factors for stroke may, in part, explain this observation.

## Strengths and limitations

Evidence is gathering as to the importance of frailty beyond traditional cardiovascular risk factors as a prognostic determinant for in patients with cardiovascular disease [13, 42], although data are lacking in evaluating this among patients with AF [12]. We present the largest study of its kind to investigate this important issue. Our estimates are adjusted for confounders informed by the existing literature. In the interests of transparency, we disclose our clinical outcome codes [43]. However, we recognise the limitations of our work. First, this work was observational and beholds inherit bias associated with such designs; whereas we report associations this does not imply causation. Moreover, a randomised controlled trial would be more appropriate methodology for the investigation of efficacy and safety of OAC in AF with frailty. Second, we had no role in the collection or processing of the raw data from constituent General Practices; coding practices may have changed over time [44]; and data were not available for calculation of bleeding scores such as HAS-BLED. Third, although recurrence of AF is more usual [45–47], some individuals with a history of AF may have had no recurrence during the study period, which may have decreased the apparent association between AF and thromboembolism. Fourth, the dataset was not linked to secondary care data, and secondary care diagnoses and investigations are potentially under-reported [48]. Fifth, OAC status was ascertained at study entry only; and although there is evidence that the prescribed dosage of DOAC is frequently incorrect [49], we did not have access to clinical data to verify dosage or evaluate contraindications. Finally, we used the full eFI in order to preserve generalisability. However, this meant that a slightly higher frailty index is inevitable in patients with AF (AF is 1 of 36 deficits).

## Conclusion

Frailty is common among older people with AF. After accounting for differences in demographics and treatment, frailty was associated with an increased risk of mortality, falls and gastrointestinal bleeding in this population. Just half the patients with an elevated stroke risk and AF were prescribed OAC, and patients with frailty were more likely to be prescribed OAC than those without frailty. Although it is possible that this represents holistic decision-making with regard to primary prevention of stroke in a population that are approaching the end of their lives, it is likely that wider use of OAC in patients at risk of stroke will reduce the burden of stroke and associated disability.

## Supplementary Material

aa-20-0775-File002_afaa265

## References

[2] Lip GYH , Tse HF, Lane DA. Atrial fibrillation. The Lancet 2012; 379: 648–61. 10.1016/S0140-6736(11)61514-6 22166900

[4] Wilke T , Groth A, Mueller S et al. Incidence and prevalence of atrial fibrillation: an analysis based on 8.3 million patients. Europace 2013; 15: 486–93. 23220354 10.1093/europace/eus333

[5] Clegg A , Young J, Iliffe S, Rikkert MO, Rockwood K. Frailty in elderly people. Lancet (London, England) 2013; 381: 752–62. 10.1016/S0140-6736(12)62167-9 PMC4098658 23395245

[6] Fumagalli S , Potpara TS, Bjerregaard Larsen T et al. Frailty syndrome: an emerging clinical problem in the everyday management of clinical arrhythmias. The results of the European Heart Rhythm Association survey. Europace 2017; 19: 1896–902. 29040554 10.1093/europace/eux288

[7] Hart RG , Pearce LA, Aguilar MI. Meta-analysis: antithrombotic therapy to prevent stroke in patients who have nonvalvular atrial fibrillation. Ann Intern Med 2007; 146: 857–67. 17577005 10.7326/0003-4819-146-12-200706190-00007

[10] National Institute for Health and Care Excellence. Multimorbidity: clinical assessment and management. In: NICE guideline NG56, 2016. https://www.nice.org.uk/guidance/ng56.

[11] Walker DM , Gale CP, Lip G et al. Editor's choice - frailty and the management of patients with acute cardiovascular disease: a position paper from the Acute Cardiovascular Care Association. Eur Heart J Acute Cardiovasc Care 2018; 7: 176–93. 29451402 10.1177/2048872618758931

[12] Wilkinson C , Todd O, Clegg A, Gale CP, Hall M. Management of atrial fibrillation for older people with frailty: a systematic review and meta-analysis. Age Ageing 2019; 48: 196–203. 30445608 10.1093/ageing/afy180 PMC6424377

[13] Farooqi MAM , Gerstein H, Yusuf S, Leong DP. Accumulation of deficits as a key risk factor for cardiovascular morbidity and mortality: a pooled analysis of 154 000 individuals. J Am Heart Assoc 2020; 9: e014686. 31986990 10.1161/JAHA.119.014686 PMC7033862

[14] Clegg A , Bates C, Young J et al. Development and validation of an electronic frailty index using routine primary care electronic health record data. Age Ageing 2016; 45: 353–60. 26944937 10.1093/ageing/afw039 PMC4846793

[15] Rockwood K , Howlett SE. Age-related deficit accumulation and the diseases of ageing. Mech Ageing Dev 2019; 180: 107–16. 31002924 10.1016/j.mad.2019.04.005

[19] Lip GY , Frison L, Halperin JL, Lane DA. Identifying patients at high risk for stroke despite anticoagulation: a comparison of contemporary stroke risk stratification schemes in an anticoagulated atrial fibrillation cohort. Stroke 2010; 41: 2731–8. 20966417 10.1161/STROKEAHA.110.590257

[20] Kirchhof P , Benussi S, Kotecha D et al. 2016 ESC guidelines for the management of atrial fibrillation developed in collaboration with EACTS. Eur Heart J 2016; 37: 2893–962. 27567408 10.1093/eurheartj/ehw210

[21] National Institute for Health and Care Excellence. Atrial fibrillation: management. CG180. 2014. https://www.nice.org.uk/guidance/cg180. 32212589

[22] Vinogradova Y , Coupland C, Hill T, Hippisley-Cox J. Risks and benefits of direct oral anticoagulants versus warfarin in a real world setting: cohort study in primary care. BMJ 2018; 362: k2505. 29973392 10.1136/bmj.k2505 PMC6031213

[24] Gordon EH , Peel NM, Samanta M, Theou O, Howlett SE, Hubbard RE. Sex differences in frailty: a systematic review and meta-analysis. Exp Gerontol 2017; 89: 30–40. 28043934 10.1016/j.exger.2016.12.021

[25] Cowan C , Healicon R, Robson I et al. The use of anticoagulants in the management of atrial fibrillation among general practices in England. Heart 2013; 99: 1166–72. 23393083 10.1136/heartjnl-2012-303472 PMC3717828

[28] Cowan JC , Wu J, Hall M, Orlowski A, West RM, Gale CP. A 10 year study of hospitalized atrial fibrillation-related stroke in England and its association with uptake of oral anticoagulation. Eur Heart J 2018; 39: 2975–83. 29982405 10.1093/eurheartj/ehy411 PMC6110195

[29] Wu J , Alsaeed ES, Barrett J, Hall M, Cowan C, Gale CP. Prescription of oral anticoagulants and antiplatelets for stroke prophylaxis in atrial fibrillation: nationwide time series ecological analysis. Europace 2020; 22: 1311–9. 32778878 10.1093/europace/euaa126 PMC7478320

[35] Wilkinson C , Cowan JC. Regional variation in anticoagulation and clinical outcomes: scope for improvement. Eur Heart J Qual Care Clin Outcomes 2018; 4: 152–4. 29912340 10.1093/ehjqcco/qcy015

[38] Alexander KP , Brouwer MA, Mulder H et al. Outcomes of apixaban versus warfarin in patients with atrial fibrillation and multi-morbidity: insights from the ARISTOTLE trial. Am Heart J 2019; 208: 123–31. 30579505 10.1016/j.ahj.2018.09.017

[39] Wilkinson C , Wu J, Searle SD et al. Clinical outcomes in patients with atrial fibrillation and frailty: insights from the ENGAGE AF-TIMI 48 trial. BMC Med. 2020. In press. doi: 10.1186/s12916-020-01869-3. PMC7758931 33357217

[40] Persky RW , Turtzo LC, McCullough LD. Stroke in women: disparities and outcomes. Curr Cardiol Rep 2010; 12: 6–13. 20425178 10.1007/s11886-009-0080-2 PMC2861793

[41] Hubbard RE , Rockwood K. Frailty in older women. Maturitas 2011; 69: 203–7. 21570783 10.1016/j.maturitas.2011.04.006

[43] Todd OM , Burton JK, Dodds RM et al. New horizons in the use of routine data for ageing research. Age Ageing 2020; 49: 716–22. 32043136 10.1093/ageing/afaa018 PMC7444666

[44] Campbell SM , Reeves D, Kontopantelis E, Sibbald B, Roland M. Effects of pay for performance on the quality of primary care in England. N Engl J Med 2009; 361: 368–78. 19625717 10.1056/NEJMsa0807651

[45] Kato T , Yamashita T, Sagara K, Iinuma H, Fu LT. Progressive nature of paroxysmal atrial fibrillation. Observations from a 14-year follow-up study. Circ J 2004; 68: 568–72. 15170094 10.1253/circj.68.568

[46] Healey JS , Connolly SJ, Gold MR et al. Subclinical atrial fibrillation and the risk of stroke. N Engl J Med 2012; 366: 120–9. 22236222 10.1056/NEJMoa1105575

[47] Lin MH , Kamel H, Singer DE, Wu YL, Lee M, Ovbiagele B. Perioperative/postoperative atrial fibrillation and risk of subsequent stroke and/or mortality. Stroke 2019; 50: 1364–71. 31043148 10.1161/STROKEAHA.118.023921

[49] Garcia Rodriguez LA , Martin-Perez M, Vora P et al. Appropriateness of initial dose of non-vitamin K antagonist oral anticoagulants in patients with non-valvular atrial fibrillation in the UK. BMJ Open 2019; 9: e031341. 10.1136/bmjopen-2019-031341 PMC6756330 31542760

