# Supplementary material for: Atrial fibrillation and oral anticoagulation in older people with frailty: a nationwide primary care electronic health records cohort study
Source: Age Ageing. 2020 Dec 16;50(3):772–9. doi: 10.1093/ageing/afaa265 (PMC8099225; doi:10.1093/ageing/afaa265)
Supplement: aa-20-0775-File002_afaa265 [file aa-20-0775-file002_afaa265.docx]

Atrial fibrillation and oral anticoagulation in older people with frailty: a nationwide primary care electronic health records cohort study.

**SUPPLEMENTARY MATERIALS**

**Contents**

[Table 1: Prevalence of atrial fibrillation by age category 2](#_Toc42536877)

[Table 2: Characteristics of patients with CHA_2_DS_2_-Vasc score of ≥2 by anticoagulation status 3](#_Toc42536878)

[Table 3A: Unadjusted association between frailty category and clinical outcomes in patients without AF, n=475,778 5](#_Toc42536879)

[Table 3B: Unadjusted association between frailty category and clinical outcomes in patients with AF, n=61,177 5](#_Toc42536880)

[Table 3C: Unadjusted association between frailty category and clinical outcomes in patients with AF and a CHA_2_DS_2_-Vasc score of two or more, n=58,204 5](#_Toc42536881)

[Table 4A: Association between frailty category and clinical outcomes in patients without AF. Adjusted for age, sex, smoking status, GP identifier and IMD at study entry. 6](#_Toc42536882)

[Table 4B: Association between frailty category and clinical outcomes in patients with AF. Adjusted for age, sex, smoking status, GP identifier and IMD at study entry. 6](#_Toc42536883)

[Table 4C: Association between frailty category and clinical outcomes in patients with AF and a CHA_2_DS_2_-Vasc score of two or more. Adjusted for age, sex, smoking status, GP identifier and IMD at study entry. 6](#_Toc42536884)

[Table 5A: Association between frailty category and clinical outcomes in patients with AF. Adjusted for age, sex, smoking status, GP identifier, IMD, prescription of antiplatelet, and prescription of OAC at study entry. 7](#_Toc42536885)

[Table 5B: Association between frailty category and clinical outcomes in patients with AF and a CHA_2_DS_2_-Vasc score of two or more. Adjusted for age, sex, smoking status, GP identifier, IMD, prescription of antiplatelet, and prescription of OAC at study entry. 7](#_Toc42536886)

[Figure 1: Strengthening the Reporting of Observational Studies in Epidemiology (STROBE) diagram of the analytical cohort. 8](#_Toc42536887)

[Figure 2: Oral anticoagulation prescription by eFI 8](#_Toc42536888)

[Full reference list 8](#_Toc42536889)

[CTV-3 code lists 10](#_Toc42536890)

Table 1: Prevalence of atrial fibrillation by age category

| Age category | n= | Patients with AF | AF prevalence, %  (95% confidence interval) | Mean CHA_2_DS_2_-Vasc, in those patients with AF (standard error) |
| --- | --- | --- | --- | --- |
| ≥65 to <70 | 169,357 | 8,391 | 5.0 (4.9 to 5.1) | 2.59 (0.132) |
| ≥70 to <75 | 127,409 | 10,463 | 8.2 (8.1 to 8.4) | 2.78 (0.122) |
| ≥75 to <80 | 98,257 | 12,721 | 13.0 (12.7 to 13.2) | 4.04 (0.115) |
| ≥80 to <85 | 72,305 | 13,215 | 18.3 (18.0 to 18.6) | 4.23 (0.119) |
| ≥85 to <90 | 45,144 | 10,194 | 22.6 (22.2 to 23.0) | 4.36 (0.134) |
| ≥90 to <95 | 19,693 | 5,046 | 25.6 (25.0 to 26.2) | 4.50 (0.192) |
| ≥95 to <100 | 4,790 | 1,147 | 23.9 (22.7 to 25.2) | 4.57 (0.389) |
| Overall | 536,955 | 61,177 | 11.4 (11.3 to 11.5) | 3.77 (0.006) |

Table 2: Characteristics of patients with CHA_2_DS_2_-Vasc score of ≥2 by anticoagulation status

|  | **Total**  **n=58,204** | | **Prescribed OAC**  **n=30,916** | | **Not prescribed OAC**  **n=27,288** | |  |  |  |
| --- | --- | --- | --- | --- | --- | --- | --- | --- | --- |
| **Demographics** |  | |  | |  | |  |  |  |
| Age, Median (IQR) | 80.2 (74.3-85.7) | | 80.1 (74.6-85.2) | | 80.5 (74.0-86.6) | |  |  |  |
| Female, n (%) | 27,987 (48.1) | | 14,285 (46.2) | | 13,702 (50.2) | |  |  |  |
| Number of eFI deficits, median (IQR) | 9 (7-12) | | 10 (7-12) | | 9 (6-12) | |  |  |  |
| IMD rank, n (%) |  | |  | |  | |  |  |  |
| Most deprived quintile | 7,188 (13.1) | | 3,654 (12.5) | | 3,534 (13.7) | |  |  |  |
| Least deprived quintile | 12,696 (23.1) | | 7,060 (24.2) | | 5,636 (21.9) | |  |  |  |
| Living in a nursing home | 5,246 (9.0) | | 2,010 (6.5) | | 3,236 (11.9) | |  |  |  |
| eFI, n (%) |  | |  | |  | |  |  |  |
| Fit | 4,863 (8.4) | | 2,028 (6.6) | | 2,835 (10.4) | |  |  |  |
| Mild | 19,198 (33.0) | | 10,221 (33.1) | | 8,977 (32.9) | |  |  |  |
| Moderate | 20,099 (34.5) | | 11,167 (36.1) | | 8,932 (32,7) | |  |  |  |
| Severe | 14,044 (24.1) | | 7,500 (24.3) | | 6,544 (24.0) | |  |  |  |
| Duration of AF, years prior to study start, Median (IQR) | 4.8 (2.2-9.4) | | 5.4 (2.4-10.1) | | 4.1 (1.9-8.4) | |  |  |  |
| CHA_2_DS_2_-Vasc, Mean (SD) | 3.9 (1.4) | | 4.0 (1.4) | | 3.8 (1.4) | |  |  |  |
| **Past medical history, n (%)** |  | |  | |  | |  |  |  |
| Alcohol excess | 1,695 (2.9) | | 789 (2.6) | | 906 (3.3) | |  |  |  |
| Anaemia | 11,974 (20.6) | | 5,959 (19.7) | | 6,015 (22.0) | |  |  |  |
| Bleeding disorder | 913 (1.6) | | 453 (1.5) | | 460 (1.7) | |  |  |  |
| Cancer | 9,862 (16.9) | | 5,045 (16.3) | | 4,817 (17.7) | |  |  |  |
| Cirrhosis | 253 (0.43) | | 111 (0.36) | | 142 (0.52) | |  |  |  |
| CKD | 20,153 (34.6) | | 11,435 (37.0) | | 8,718 (32.0) | |  |  |  |
| Falls | 11,637 (20.0) | | 5,843 (18.9) | | 5,794 (21.2) | |  |  |  |
| GI bleed |  | |  | |  | |  |  |  |
| Upper | 890 (1.5) | | 403 (1.3) | | 487 (1.8) | |  |  |  |
| Lower | 5,940 (10.2) | | 3,075 (10.0) | | 2,865 (10.5) | |  |  |  |
| Unspecified | 524 (0.90) | | 228 (0.74) | | 296(1.1) | |  |  |  |
| Haematuria | 7,245 (12.5) | | 4,116 (13.3) | | 3,129 (11.5) | |  |  |  |
| Haemoptysis | 1,705 (2.9) | | 999 (3.2) | | 706 (2.6) | |  |  |  |
| Heart failure | 12,320 (21.2) | | 8,016 (25.9) | | 4,304 (15.8) | |  |  |  |
| Hypertension | 41,146 (70.7) | | 22,173 (71.7) | | 18,991 (69.6) | |  |  |  |
| Hyperthyroidism | 1,944 (3.3) | | 1,046 (3.4) | | 898 (3.3) | |  |  |  |
| Intracranial bleeding | 972 (1.7) | | 303 (0.98) | | 669 (2.5) | |  |  |  |
| Ischaemic heart disease | 19,158 (32.9) | | 10,741 (34.7) | | 8,417 (30.9) | |  |  |  |
| Memory loss | 7,782 (13.4) | | 3,474 (11.2) | | 4,308 (15.8) | |  |  |  |
| Myocardial infarction | 7,400 (12.7) | | 3,939 (12.7) | | 3,461 (12.7) | |  |  |  |
| Obesity | 818 (1.4) | | 481 (1.6) | | 337 (1.2) | |  |  |  |
| Peptic ulcer | 3,555 (6.1) | | 1,723 (5.6) | | 1,832 (6.7) | |  |  |  |
| Varices | 82 (0.14) | | 28 (0.09) | | 54 (0.20) | |  |  |  |
| Stroke | 7,239 (12.4) | | 4,375 (14.2) | | 2,864 (10.5) | |  |  |  |
| Ischaemic | 3,179 (5.5) | | 1,983 (6.4) | | 1,196 (4.4) | |  |  |  |
| Unspecified | 5,043 (8.7) | | 3,003 (9.7) | | 2,040 (7.5) | |  |  |  |
| Transient ischaemic attack | 6,019 (10.3) | | 3,624 (11.7) | | 2,395 (8.8) | |  |  |  |
| Pulmonary embolism | 1,820 (3.1) | | 1,312 (4.2) | | 508 (1.9) | |  |  |  |
| Deep vein thrombosis | 2,264 (3.9) | | 1,413 (4.6) | | 851 (3.1) | |  |  |  |
| **Medications in the previous year, n (%)** | | | |  | |  | | |  |
| Proton pump inhibitor | | 23,695 (40.7) | | 11,852 (38.3) | | 11,843 (43.4) | | |  |
| Macrolide antibiotics | | 402 (0.69) | | 196 (0.63) | | 206 (0.75) | | |  |
| NSAID | | 5,209 (9.0) | | 2,288 (7.4) | | 2,921 (10.7) | | |  |
| Corticosteroid | | 1,684 (2.9) | | 901 (2.9) | | 783 (2.9) | | |  |
| Statin | | 35,236 (60.5) | | 19,972 (64.6) | | 15,264 (55.9) | | |  |
| Anti-epileptic | |  | |  | |  | | |  |
| Carbemazepine | | 224 (0.38) | | 111 (0.36) | | 113 (0.41) | | |  |
| Phenytoin | | 176 (0.30) | | 85 (0.27) | | 91 (0.33) | | |  |
| **Medication at study entry, n (%)** | | | |  | |  | | |  |
| Any anti-platelet | | 3,688 (6.3) | | 644 (2.1) | | 3,044 (11.2) | | |  |
| Missing IMD data: 3,252 (5.6%)  **Abbreviations:** AF: atrial fibrillation; CHA_2_DS_2_-Vasc: one point for age 65-74 years, female sex; history of heart failure, hypertension, vascular disease, or diabetes. Two points are allocated for age >75 years, and two points for a history of stroke, transient ischaemic attack or thromboembolism. eFI: electronic frailty index; IMD: indices of multiple deprivation; IQR: interquartile range; n: number; OAC: oral anticoagulation; SD: standard deviation | | | | | | | |  |  |

Table 3A: Unadjusted association between frailty category and clinical outcomes in patients without AF, n=475,778

|  | Fit | Mild, OR (95% CI) | Moderate, OR (95% CI) | Severe, OR (95% CI) |
| --- | --- | --- | --- | --- |
| Mortality | REF | 2.74 (2.62 - 2.87) | 6.21 (5.94 - 6.50) | 9.99 (9.52 - 10.49) |
| Stroke | REF | 2.58 (2.20 - 3.02) | 5.12 (4.35 - 6.03) | 7.54 (6.29 - 9.04) |
| Gastrointestinal bleed | REF | 1.68 (1.53 - 2.85) | 2.46 (2.21 - 2.73) | 2.51 (2.18 - 2.89) |
| Intracranial bleed | REF | 1.81 (1.45 - 2.25) | 2.47 (1.92 - 3.17) | 2.86 (2.09 - 3.91) |
| Falls | REF | 3.75 (3.38 – 4.16) | 10.38 (9.38 – 11.48) | 20.08 (18.10 – 22.28) |
| Transient ischaemic attack | REF | 1.64 (1.47 - 1.83) | 2.65 (2.35 - 2.99) | 2.82 (2.41 - 3.29) |

**Abbreviations** CI: confidence interval; n: number; OR: odds ratio; REF: reference

Table 3B: Unadjusted association between frailty category and clinical outcomes in patients with AF, n=61,177

|  | Fit | Mild, OR (95% CI) | Moderate, OR (95% CI) | Severe, OR (95% CI) |
| --- | --- | --- | --- | --- |
| Mortality | REF | 2.04 (1.73 - 2.40) | 4.21 (3.59 - 4.93) | 8.71 (7.44 - 10.19) |
| Stroke | REF | 1.28 (0.79 - 2.07) | 2.43 (1.54 - 3.84) | 3.30 (2.08 - 5.22) |
| Gastrointestinal bleed | REF | 1.29 (0.90 - 1.86) | 2.00 (1.40 - 2.84) | 2.60 (1.82 - 3.71) |
| Intracranial bleed | REF | 0.99 (0.49 - 2.03) | 1.52 (0.77 - 3.02) | 2.48 (1.26 - 4.90) |
| Falls | REF | 4.40 (2.51 – 7.73) | 11.20 (6.45 – 19.44) | 31.25 (18.06 – 54.06) |
| Transient ischaemic attack | REF | 1.15 (0.74 - 1.77) | 1.70 (1.12 - 2.59) | 2.30 (1.50 - 3.51) |

**Abbreviations** CI: confidence interval; n: number; OR: odds ratio; REF: reference

Table 3C: Unadjusted association between frailty category and clinical outcomes in patients with AF and a CHA_2_DS_2_-Vasc score of two or more, n=58,204

|  | Fit | Mild, OR (95% CI) | Moderate, OR (95% CI) | Severe, OR (95% CI) |
| --- | --- | --- | --- | --- |
| Mortality | REF | 1.88 (1.57 - 2.24) | 3.78 (3.18 - 4.48) | 7.76 (6.54 -9.20) |
| Stroke | REF | 1.22 (0.72 - 2.06) | 2.26 (1.37 - 3.73) | 3.07 (1.86 - 5.09) |
| Gastrointestinal bleed | REF | 1.06 (0.73 - 1.56) | 1.66 (1.15 - 2.39) | 2.14 (1.48 - 3.10) |
| Intracranial bleed | REF | 0.83 (0.39 - 1.75) | 1.29 (0.63 - 2.63) | 2.08 (1.02 - 4.24) |
| Falls | REF | 3.36 (1.91 – 5.91) | 8.46 (4.87 – 14.68) | 23.57 (13.62 – 40.77) |
| Transient ischaemic attack | REF | 1.00 (0.63 - 1.60) | 1.53 (0.98 - 2.40) | 2.05 (1.30 - 3.23) |

**Abbreviations** CI: confidence interval; n: number; OR: odds ratio; REF: reference

Table 4A: Association between frailty category and clinical outcomes in patients without AF. Adjusted for age, sex, smoking status, GP identifier and IMD at study entry.

|  | Fit | Mild, OR (95% CI) | Moderate, OR (95% CI) | Severe, OR (95% CI) |
| --- | --- | --- | --- | --- |
| Mortality | REF | 1.87 (1.79 - 1.96) | 3.02 (2.84 - 3.17) | 4.31 (4.08 - 4.55) |
| Stroke | REF | 1.77 (1.50 - 2.09) | 2.57 (2.15 - 3.07) | 3.22 (2.63 - 3.95) |
| Gastrointestinal bleed | REF | 1.63 (1.48 - 1.80) | 2.35 (2.09 - 2.64) | 2.41 (2.07 - 2.80) |
| Intracranial bleed | REF | 1.42 (1.12 - 1.78) | 1.63 v(1.24 - 2.15) | 1.85 (1.32 - 2.60) |
| Falls | REF | 2.29 (2.05 - 2.55) | 4.17 (3.73 – 4.65) | 6.54 (5.82 – 7.34) |
| Transient ischaemic attack | REF | 1.42 (1.26 - 1.59) | 1.97 (1.73 - 2.26) | 2.00 (1.68 - 2.37) |

**Abbreviations** CI: confidence interval; n: number; OR: odds ratio; REF: reference

Table 4B: Association between frailty category and clinical outcomes in patients with AF. Adjusted for age, sex, smoking status, GP identifier and IMD at study entry.

|  | Fit | Mild, OR (95% CI) | Moderate, OR (95% CI) | Severe, OR (95% CI) |
| --- | --- | --- | --- | --- |
| Mortality | REF | 1.49 (1.26 - 1.76) | 2.41 (2.04 - 2.84) | 4.01 (3.40 - 4.73) |
| Stroke | REF | 0.98 (0.60 - 1.60) | 1.44 (0.89 - 2.31) | 1.47 (0.90 - 2.41) |
| Gastrointestinal bleed | REF | 2.32 (0.90 - 1.94) | 2.02 (1.38 - 2.94) | 2.71 (1.84 - 4.01) |
| Intracranial bleed | REF | 0.86 (0.40 - 1.82) | 1.11 (0.53 - 2.34) | 1.63 (0.76 - 3.47) |
| Falls | REF | 2.92 (1.66 – 5.13) | 5.14 (2.95 – 8.96) | 10.26 (5.90 – 17.86) |
| Transient ischaemic attack | REF | 1.02 (0.65 - 1.60) | 1.45 (0.93 - 2.25) | 1.76 (1.11 - 2.79) |

**Abbreviations** CI: confidence interval; n: number; OR: odds ratio; REF: reference

Table 4C: Association between frailty category and clinical outcomes in patients with AF and a CHA_2_DS_2_-Vasc score of two or more. Adjusted for age, sex, smoking status, GP identifier and IMD at study entry.

|  | Fit | Mild, OR (95% CI) | Moderate, OR (95% CI) | Severe, OR (95% CI) |
| --- | --- | --- | --- | --- |
| Mortality | REF | 1.48 (1.23 - 1.77) | 2.38 (1.99 - 2.84) | 3.97 (3.32 - 4.74) |
| Stroke | REF | 0.99 (0.58 - 1.70) | 1.45 (0.86 - 2.44) | 1.49 (0.87 - 2.55) |
| Gastrointestinal bleed | REF | 1.11 (0.74 - 1.64) | 1.72 (1.17 - 2.54) | 2.31 (1.54 - 3.44) |
| Intracranial bleed | REF | 0.75 (0.34 - 1.66) | 1.02 (0.47 - 2.20) | 1.48 (0.68 - 3.23) |
| Falls | REF | 2.42 (1.38 – 4.26) | 4.33 (2.49 – 7.54) | 8.64 (4.97 – 15.03) |
| Transient ischaemic attack | REF | 0.88 (0.55 - 1.41) | 1.30 (0.98 - 2.06) | 1.57 (0.98 - 2.53) |

**Abbreviations** CI: confidence interval; n: number; OR: odds ratio; REF: reference

Table 5A: Association between frailty category and clinical outcomes in patients with AF. Adjusted for age, sex, smoking status, GP identifier, IMD, prescription of antiplatelet, and prescription of OAC at study entry.

|  | Fit | Mild, OR (95% CI) | Moderate, OR (95% CI) | Severe, OR (95% CI) |
| --- | --- | --- | --- | --- |
| Mortality | REF | 1.48 (1.23 - 1.77) | 2.38 (1.99 - 2.84) | 3.97 (3.32 - 4.74) |
| Stroke | REF | 0.99 (0.58 - 1.70) | 1.45 (0.86 - 2.44) | 1.49 (0.87 - 2.55) |
| Gastrointestinal bleed | REF | 1.11 (0.74 - 1.64) | 1.72 (1.17 - 2.54) | 2.31 (1.54 - 3.44) |
| Intracranial bleed | REF | 0.75 (0.34 - 1.66) | 1.02 (0.47 - 2.20) | 1.48 (0.68 - 3.23) |
| Falls | REF | 2.90 (1.65 – 5.11) | 5.12 (2.94 – 8.94) | 10.25 (5.88 – 17.85) |
| Transient ischaemic attack | REF | 0.88 (0.55 - 1.41) | 1.30 (0.98 - 2.06) | 1.57 (0.98 - 2.53) |

**Abbreviations** CI: confidence interval; n: number; OR: odds ratio; REF: reference

Table 5B: Association between frailty category and clinical outcomes in patients with AF and a CHA_2_DS_2_-Vasc score of two or more. Adjusted for age, sex, smoking status, GP identifier, IMD, prescription of antiplatelet, and prescription of OAC at study entry.

|  | Fit | Mild, OR (95% CI) | Moderate, OR (95% CI) | Severe, OR (95% CI) |
| --- | --- | --- | --- | --- |
| Mortality | REF | 1.52 (1.27-1.82) | 2.47 (2.06 - 2.94) | 4.09 (3.43 - 4.89) |
| Stroke | REF | 1.00 (0.58 - 1.72) | 1.44 (0.85 - 2.43) | 1.45 (0.85 - 2.48) |
| Gastrointestinal bleed | REF | 1.10 (0.74 - 1.64) | 1.71 (1.16 - 2.53) | 2.30 (1.54 - 3.45) |
| Intracranial bleed | REF | 0.72 (0.33 - 1.59) | 0.96 (0.44 - 2.08) | 1.40 (0.64 - 3.07) |
| Falls | REF | 2.41 (1.37 – 4.25) | 4.31 (2.48 – 7.51) | 8.62 (4.96 – 15.00) |
| Transient ischaemic attack | REF | 0.87 (0.54 - 1.39) | 1.25 (0.78 - 1.98) | 1.46 (0.90 - 2.35) |

**Abbreviations** CI: confidence interval; n: number; OR: odds ratio; REF: reference

Figure 1: Strengthening the Reporting of Observational Studies in Epidemiology (STROBE) diagram of the analytical cohort.


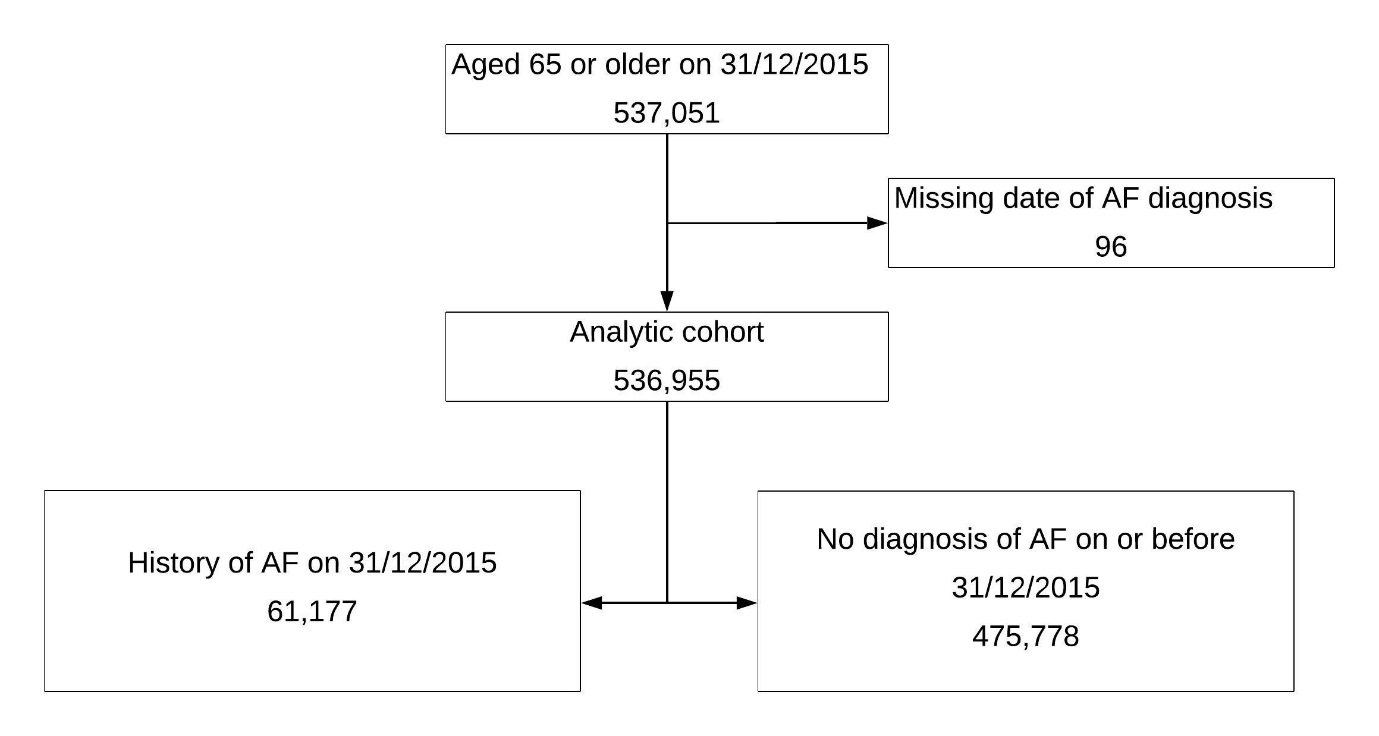


Figure 2: Oral anticoagulation prescription by eFI


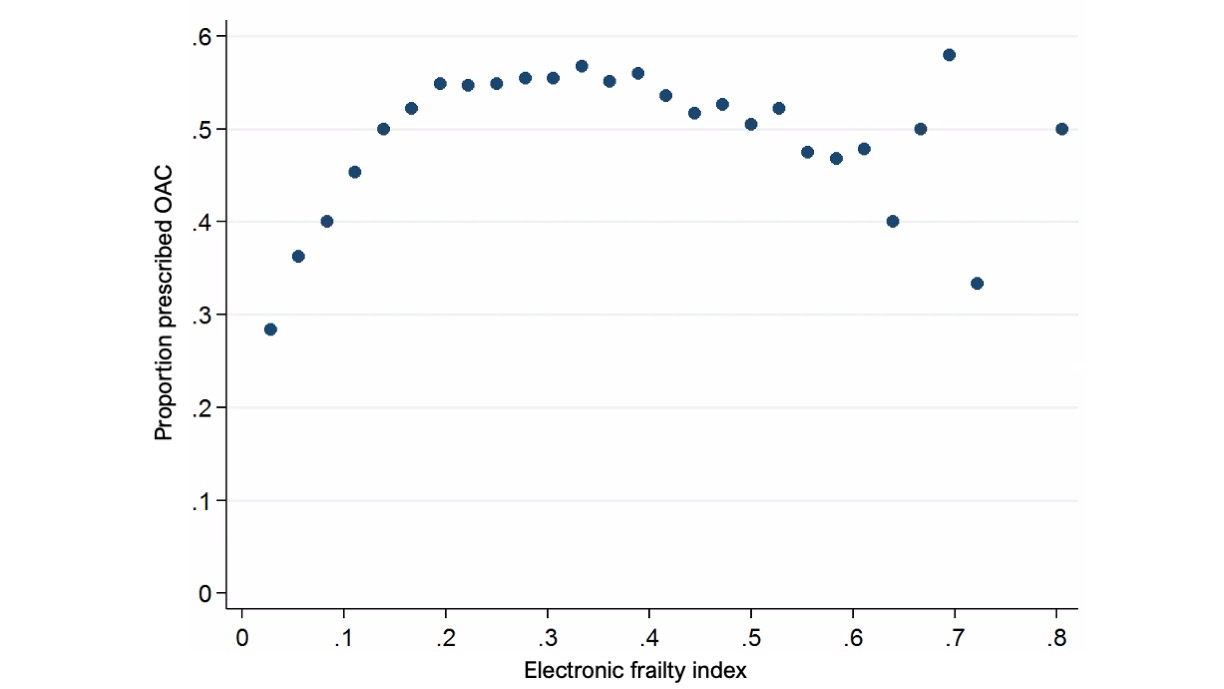


Full reference list

1. Timmis A, Townsend N, Gale CP, Torbica A, Lettino M, Petersen SE, et al. European Society of Cardiology: Cardiovascular Disease Statistics 2019. Eur Heart J. 2020 Jan 1;41(1):12-85.

2. Lip GYH, Tse HF, Lane DA. Atrial fibrillation. The Lancet. 2012;379(9816):648-61.

3. Hobbs FD, Fitzmaurice DA, Mant J, Murray E, Jowett S, Bryan S, et al. A randomised controlled trial and cost-effectiveness study of systematic screening (targeted and total population screening) versus routine practice for the detection of atrial fibrillation in people aged 65 and over. The SAFE study. Health technology assessment (Winchester, England). 2005 Oct;9(40):iii-iv, ix-x, 1-74.

4. Wilke T, Groth A, Mueller S, Pfannkuche M, Verheyen F, Linder R, et al. Incidence and prevalence of atrial fibrillation: an analysis based on 8.3 million patients. Europace. 2013 Apr;15(4):486-93.

5. Clegg A, Young J, Iliffe S, Rikkert MO, Rockwood K. Frailty in elderly people. Lancet (London, England). 2013 Mar 2;381(9868):752-62.

6. Fumagalli S, Potpara TS, Bjerregaard Larsen T, Haugaa KH, Dobreanu D, Proclemer A, et al. Frailty syndrome: an emerging clinical problem in the everyday management of clinical arrhythmias. The results of the European Heart Rhythm Association survey. Europace. 2017 Nov 1;19(11):1896-902.

7. Hart RG, Pearce LA, Aguilar MI. Meta-analysis: antithrombotic therapy to prevent stroke in patients who have nonvalvular atrial fibrillation. Annals of internal medicine. 2007 Jun 19;146(12):857-67.

8. Lip GY, Clementy N, Pericart L, Banerjee A, Fauchier L. Stroke and major bleeding risk in elderly patients aged >/=75 years with atrial fibrillation: the Loire Valley atrial fibrillation project. Stroke. 2015 Jan;46(1):143-50.

9. Monette J, Gurwitz JH, Rochon PA, Avorn J. Physician attitudes concerning warfarin for stroke prevention in atrial fibrillation: results of a survey of long-term care practitioners. J Am Geriatr Soc. 1997 Sep;45(9):1060-5.

10. National Institute for Health and Care Excellence. Multimorbidity: clinical assessment and management. NICE guideline NG56. 2016.

11. Walker DM, Gale CP, Lip G, Martin-Sanchez FJ, McIntyre HF, Mueller C, et al. Editor's Choice - Frailty and the management of patients with acute cardiovascular disease: A position paper from the Acute Cardiovascular Care Association. European heart journal Acute cardiovascular care. 2018 Mar;7(2):176-93.

12. Wilkinson C, Todd O, Clegg A, Gale CP, Hall M. Management of atrial fibrillation for older people with frailty: a systematic review and meta-analysis. Age Ageing. 2019 Mar 1;48(2):196-203.

13. Farooqi MAM, Gerstein H, Yusuf S, Leong DP. Accumulation of Deficits as a Key Risk Factor for Cardiovascular Morbidity and Mortality: A Pooled Analysis of 154 000 Individuals. J Am Heart Assoc. 2020 Feb 4;9(3):e014686.

14. Clegg A, Bates C, Young J, Ryan R, Nichols L, Ann Teale E, et al. Development and validation of an electronic frailty index using routine primary care electronic health record data. Age Ageing. 2016 May;45(3):353-60.

15. Rockwood K, Howlett SE. Age-related deficit accumulation and the diseases of ageing. Mech Ageing Dev. 2019 Jun;180:107-16.

16. Brundle C, Heaven A, Brown L, Teale E, Young J, West R, et al. Convergent validity of the electronic frailty index. Age Ageing. 2019 Jan 1;48(1):152-6.

17. Kim SW, Yoon SJ, Choi JY, Kang MG, Cho Y, Oh IY, et al. Clinical implication of frailty assessment in older patients with atrial fibrillation. Arch Gerontol Geriatr. 2017 May - Jun;70:1-7.

18. Kontopantelis E, Stevens RJ, Helms PJ, Edwards D, Doran T, Ashcroft DM. Spatial distribution of clinical computer systems in primary care in England in 2016 and implications for primary care electronic medical record databases: a cross-sectional population study. BMJ Open. 2018 Feb 28;8(2):e020738.

19. Lip GY, Frison L, Halperin JL, Lane DA. Identifying patients at high risk for stroke despite anticoagulation: a comparison of contemporary stroke risk stratification schemes in an anticoagulated atrial fibrillation cohort. Stroke. 2010 Dec;41(12):2731-8.

20. Kirchhof P, Benussi S, Kotecha D, Ahlsson A, Atar D, Casadei B, et al. 2016 ESC Guidelines for the management of atrial fibrillation developed in collaboration with EACTS. Eur Heart J. 2016 Oct 7;37(38):2893-962.

21. National Institute for Health and Care Excellence. Atrial fibrillation: management. CG180. 2014.

22. Vinogradova Y, Coupland C, Hill T, Hippisley-Cox J. Risks and benefits of direct oral anticoagulants versus warfarin in a real world setting: cohort study in primary care. BMJ. 2018 Jul 4;362:k2505.

23. Fine JP, Gray RJ. A Proportional Hazards Model for the Subdistribution of a Competing Risk. Journal of the American Statistical Association. 1999;94(446):496-509.

24. Gordon EH, Peel NM, Samanta M, Theou O, Howlett SE, Hubbard RE. Sex differences in frailty: A systematic review and meta-analysis. Exp Gerontol. 2017 Mar;89:30-40.

25. Cowan C, Healicon R, Robson I, Long WR, Barrett J, Fay M, et al. The use of anticoagulants in the management of atrial fibrillation among general practices in England. Heart. 2013 Aug;99(16):1166-72.

26. Holt TA, Hunter TD, Gunnarsson C, Khan N, Cload P, Lip GY. Risk of stroke and oral anticoagulant use in atrial fibrillation: a cross-sectional survey. Br J Gen Pract. 2012 Oct;62(603):e710-7.

27. Christesen AMS, Vinter N, Mortensen LS, Fenger-Gron M, Johnsen SP, Frost L. Inequality in oral anticoagulation use and clinical outcomes in atrial fibrillation: a Danish nationwide perspective. Eur Heart J Qual Care Clin Outcomes. 2018 Jul 1;4(3):189-99.

28. Cowan JC, Wu J, Hall M, Orlowski A, West RM, Gale CP. A 10 year study of hospitalized atrial fibrillation-related stroke in England and its association with uptake of oral anticoagulation. Eur Heart J. 2018 Aug 21;39(32):2975-83.

29. Wu J, Alsaeed ES, Barrett J, Hall M, Cowan C, Gale CP. Prescription of oral anticoagulants and antiplatelets for stroke prophylaxis in atrial fibrillation: nationwide time series ecological analysis. Europace. 2020 Sep 1;22(9):1311-9.

30. Feigin VL, Forouzanfar MH, Krishnamurthi R, Mensah GA, Connor M, Bennett DA, et al. Global and regional burden of stroke during 1990-2010: findings from the Global Burden of Disease Study 2010. Lancet (London, England). 2014 Jan 18;383(9913):245-54.

31. Steiner T, Weitz JI, Veltkamp R. Anticoagulant-Associated Intracranial Hemorrhage in the Era of Reversal Agents. Stroke. 2017 May;48(5):1432-7.

32. Hagerty T, Rich MW. Fall risk and anticoagulation for atrial fibrillation in the elderly: A delicate balance. Cleve Clin J Med. 2017 Jan;84(1):35-40.

33. Spaniolas K, Cheng JD, Gestring ML, Sangosanya A, Stassen NA, Bankey PE. Ground level falls are associated with significant mortality in elderly patients. J Trauma. 2010 Oct;69(4):821-5.

34. Vallakati A, Lewis WR. Underuse of anticoagulation in patients with atrial fibrillation. Postgrad Med. 2016 2016/02/17;128(2):191-200.

35. Wilkinson C, Cowan JC. Regional variation in anticoagulation and clinical outcomes: scope for improvement. Eur Heart J Qual Care Clin Outcomes. 2018 Jul 1;4(3):152-4.

36. Frewen J, Finucane C, Cronin H, Rice C, Kearney PM, Harbison J, et al. Factors that influence awareness and treatment of atrial fibrillation in older adults. QJM. 2013 May;106(5):415-24.

37. Madhavan M, Holmes DN, Piccini JP, Ansell JE, Fonarow GC, Hylek EM, et al. Association of frailty and cognitive impairment with benefits of oral anticoagulation in patients with atrial fibrillation. Am Heart J. 2019 May;211:77-89.

38. Alexander KP, Brouwer MA, Mulder H, Vinereanu D, Lopes RD, Proietti M, et al. Outcomes of apixaban versus warfarin in patients with atrial fibrillation and multi-morbidity: Insights from the ARISTOTLE trial. Am Heart J. 2019 Feb;208:123-31.

39. Wilkinson C, Wu J, Searle SD, Todd OM, Hall M, Kunadian V, et al. Clinical outcomes in patients with atrial fibrillation and frailty: insights from the ENGAGE AF-TIMI 48 trial. BMC Medicine. 2020:IN REVIEW.

40. Persky RW, Turtzo LC, McCullough LD. Stroke in women: disparities and outcomes. Curr Cardiol Rep. 2010 Jan;12(1):6-13.

41. Hubbard RE, Rockwood K. Frailty in older women. Maturitas. 2011 Jul;69(3):203-7.

42. Dewan P, Jackson A, Jhund PS, Shen L, Ferreira JP, Petrie MC, et al. The prevalence and importance of frailty in heart failure with reduced ejection fraction – an analysis of PARADIGM-HF and ATMOSPHERE. European Journal of Heart Failure.n/a(n/a).

43. Todd OM, Burton JK, Dodds RM, Hollinghurst J, Lyons RA, Quinn TJ, et al. New horizons in the use of routine data for ageing research. Age and Ageing. 2020.

44. Campbell SM, Reeves D, Kontopantelis E, Sibbald B, Roland M. Effects of pay for performance on the quality of primary care in England. N Engl J Med. 2009 Jul 23;361(4):368-78.

45. Kato T, Yamashita T, Sagara K, Iinuma H, Fu LT. Progressive nature of paroxysmal atrial fibrillation. Observations from a 14-year follow-up study. Circ J. 2004 Jun;68(6):568-72.

46. Healey JS, Connolly SJ, Gold MR, Israel CW, Van Gelder IC, Capucci A, et al. Subclinical atrial fibrillation and the risk of stroke. N Engl J Med. 2012 Jan 12;366(2):120-9.

47. Lin MH, Kamel H, Singer DE, Wu YL, Lee M, Ovbiagele B. Perioperative/Postoperative Atrial Fibrillation and Risk of Subsequent Stroke and/or Mortality. Stroke. 2019 Jun;50(6):1364-71.

48. Woodfield R, Grant I, Group UKBSO, Follow-Up UKB, Outcomes Working G, Sudlow CL. Accuracy of Electronic Health Record Data for Identifying Stroke Cases in Large-Scale Epidemiological Studies: A Systematic Review from the UK Biobank Stroke Outcomes Group. PLoS One. 2015;10(10):e0140533.

49. Garcia Rodriguez LA, Martin-Perez M, Vora P, Roberts L, Balabanova Y, Brobert G, et al. Appropriateness of initial dose of non-vitamin K antagonist oral anticoagulants in patients with non-valvular atrial fibrillation in the UK. BMJ Open. 2019 Sep 20;9(9):e031341.

CTV-3 code lists

Atrial fibrillation or flutter were defined by the following CTV-3 codes:

| 3272 | ECG: atrial fibrillation |
| --- | --- |
| G5730 | Atrial fibrillation |
| 2432 | O/E - pulse irregularly irreg. |
| XaOfa | Persistent atrial fibrillation |
| XaIIT | Atrial fibrillation monitoring |
| XaMGD | Atrial fibrillation annual review |
| XaLFj | Excepted from atrial fibrillation qual indic: Inform dissent |
| XaOft | Permanent atrial fibrillation |
| XaDv6 | H/O: atrial fibrillation |
| Xa2E8 | Paroxysmal atrial fibrillation |
| G5731 | Atrial flutter |
| Xa7nI | Controlled atrial fibrillation |
| X202R | Lone atrial fibrillation |
| XaLFz | Atrial fibrillation resolved |
| XaEga | Rapid atrial fibrillation |
| G573. | Atrial fibrillation and flutter |
| XaLFi | Except from atr fib quality indicators: Patient unsuitable |
| 3273 | ECG: atrial flutter |
| XaaUH | Paroxysmal atrial flutter |
| XE0Wk | (Atrial fibrillation) or (atrial flutter) |
| G573z | Atrial fibrillation and flutter NOS |
| XaMDG | Atrial fibrillation monitoring first letter |
| XaXrZ | Referral to atrial fibrillation clinic |
| XaeUP | Chronic atrial fibrillation |
| XaNRA | History of atrial flutter |
| XaLFh | Exception reporting: atrial fibrillation quality indicators |
| XaMFn | Atrial fibrillation monitoring telephone invite |
| XaeUQ | Typical atrial flutter |
| XaMDF | Atrial fibrillation monitoring administration |
| XaMDH | Atrial fibrillation monitoring second letter |
| XaMDI | Atrial fibrillation monitoring third letter |
| X202S | Non-rheumatic atrial fibrillation |
| 7936A | Implant intravenous pacemaker for atrial fibrillation |
| XaZdc | Atrial fibrillation care pathway |
| XaMDK | Atrial fibrillation monitoring verbal invite |
| XaeUR | Atypical atrial flutter |

The following CTV-3 codes defined the outcomes and featured in the ResearchOne extract:

| Falls | 16D.. | Falls |
| --- | --- | --- |
|  | TC... | Accidental fall |
|  | Xa1GP | Recurrent falls |
|  | Xa6uH | Elderly fall |
|  | TCz.. | Accidental falls NOS |
|  | TC5.. | Fall on same level from slipping, tripping or stumbling |
|  | XaLqJ | Referral to falls service |
|  | Xa6uG | Observation of falls |
|  | Y3356 | Unable to get off floor |
|  |  |  |
| Gastrointestinal bleed | J5730 | Rectal haemorrhage |
|  | X30Bj | Bleeding per rectum |
|  | XaJuv | Painless rectal bleeding |
|  | J573. | (Haemorrhage of rectum & anus) or (PR - bleeding per rectum) |
|  | XE0d3 | Anal &/or rectal haemorrhage |
|  | XaJuu | Painful rectal bleeding |
|  | G8480 | Bleeding haemorrhoids NOS |
|  | X76fy | Bleeding pile |
|  | J5731 | Anal haemorrhage |
|  | X30Bk | Fresh blood passed per rectum |
|  | G8450 | External bleeding haemorrhoids |
|  | X76fR | Bleeding from anus |
|  | X30Bi | Lower gastrointestinal haemorrhage |
|  | G8420 | Internal bleeding haemorrhoids |
|  | XE0b0 | Haemorrhage of rectum and anus |
|  | J573z | Haemorrhage of rectum and anus NOS |
|  | X30Ct | Stomal bleeding |
|  | J68.. | Gastrointestinal haemorrhage |
|  | XaB3J | Recurrent gastrointestinal bleeding |
|  | XaB3K | Massive gastrointestinal bleed |
|  | J68z. | Gastrointestinal bleeding (& [unspecified]) |
|  | XE0bJ | Gastrointestinal haemorrhage unspecified |
|  | J68z1 | Intestinal haemorrhage NOS |
|  | J68zz | Gastrointestinal tract haemorrhage NOS |
|  | Xa00e | Sepsis-associated gastrointestinal haemorrhage |
|  | J680. | Haematemesis |
|  | XE0rB | Vomiting blood - fresh |
|  | X30Bh | Bleeding duodenal ulcer |
|  | X30Be | Upper gastrointestinal haemorrhage |
|  | XaBfG | Haematemesis - cause unknown |
|  | G850. | Bleeding oesophageal varices |
|  | J1201 | Acute duodenal ulcer with haemorrhage |
|  | J68z0 | Gastric haemorrhage NOS |
|  | J1211 | Chronic duodenal ulcer with haemorrhage |
|  | X30Bg | Bleeding gastric ulcer |
|  | J1101 | Acute gastric ulcer with haemorrhage |
|  | J1111 | Chronic gastric ulcer with haemorrhage |
|  | XaB5h | Haemorrhagic oesophagitis |
|  | J11y1 | Unspecified gastric ulcer with haemorrhage |
|  | Xa7TU | Oesophageal bleeding |
|  | J1103 | Acute gastric ulcer with haemorrhage and perforation |
|  | Xa363 | Vomiting stale blood |
|  | J1113 | Chronic gastric ulcer with haemorrhage and perforation |
|  | XaBel | Bleeding stress ulcer of stomach |
|  | J11y3 | Unspecified gastric ulcer with haemorrhage and perforation |
|  | 760J4 | Balloon tamponade of oesophagus |
|  |  |  |
| Intracranial haemorrhage | G613. | Cerebellar haemorrhage |
|  | G61z. | Intracerebral haemorrhage NOS |
|  | XM0rV | Cerebral haemorrhage |
|  | XE0VF | Cerebral parenchymal haemorrhage |
|  | Gyu6F | [X]Intracerebral haemorrhage in hemisphere, unspecified |
|  | XaBM4 | Left sided intracerebral haemorrhage, unspecified |
|  | X00DQ | Brainstem haemorrhage |
|  | G614. | Pontine haemorrhage |
|  | X00DO | Thalamic haemorrhage |
|  | XE0Wy | Cerebral haemorrhage NOS |
|  | G612. | Basal ganglia haemorrhage |
|  | X00DP | Lacunar haemorrhage |
|  | G611. | Internal capsule haemorrhage |
|  | G617. | Intracerebral haemorrhage, intraventricular |
|  | XaBM5 | Right sided intracerebral haemorrhage, unspecified |
|  | G610. | Cortical haemorrhage |
|  | X00DM | Lobar cerebral haemorrhage |
|  | G616. | External capsule haemorrhage |
|  | X00DN | Subcortical cerebral haemorrhage |
|  | G618. | Intracerebral haemorrhage, multiple localised |
|  | G615. | Bulbar haemorrhage |
|  | G613. | Cerebellar haemorrhage |
|  | G61z. | Intracerebral haemorrhage NOS |
|  | G61.. | Intracerebral haemorrhage (& [cerebrovasc accident due to]) |
|  | XE0VF | Cerebral parenchymal haemorrhage |
|  | XaBM4 | Left sided intracerebral haemorrhage, unspecified |
|  | X00DQ | Brainstem haemorrhage |
|  | G614. | Pontine haemorrhage |
|  | X00DO | Thalamic haemorrhage |
|  | G612. | Basal ganglia haemorrhage |
|  | X00DP | Lacunar haemorrhage |
|  | G611. | Internal capsule haemorrhage |
|  | XaBM5 | Right sided intracerebral haemorrhage, unspecified |
|  | G610. | Cortical haemorrhage |
|  | X00DM | Lobar cerebral haemorrhage |
|  | G616. | External capsule haemorrhage |
|  | X00DN | Subcortical cerebral haemorrhage |
|  | G618. | Intracerebral haemorrhage, multiple localised |
|  | G615. | Bulbar haemorrhage |
|  | Xa0AB | Subdural haematoma |
|  |  |  |
| Stroke - infarct | Xa00I | Occipital cerebral infarction |
|  | X00DA | Lacunar infarction |
|  | Xa0kZ | Cerebral infarction |
|  | X00DI | Haemorrhagic cerebral infarction |
|  | X00D8 | Posterior cerebral circulation infarction |
|  | X00D7 | Partial anterior cerebral circulation infarction |
|  | Xa00K | Brainstem infarction |
|  | G640. | Cerebral thrombosis |
|  | X00D3 | CVA - cerebral artery occlusion |
|  | X00D6 | Total anterior cerebral circulation infarction |
|  | Xa00J | Cerebellar infarction |
|  | XaBED | Right sided cerebral infarction |
|  | XaBEC | Left sided cerebral infarction |
|  | XaJgQ | Infarction of basal ganglia |
|  | XaB4Z | Multiple lacunar infarcts |
|  | XE0VJ | Cerebral infarction NOS |
|  | X00DC | Pure sensory lacunar infarction |
|  | XaQbK | Pure motor lacunar syndrome |
|  | X00D5 | Anterior cerebral circulation infarction |
|  | G6410 | Cerebral infarction due to embolism of cerebral arteries |
|  | G64z. | Infarct (& [cerebell] or [cerebral NOS] or [brainstem NOS]) |
|  | Gyu64 | [X]Other cerebral infarction |
|  | G6400 | Cerebral infarction due to thrombosis of cerebral arteries |
|  | X00D9 | Brainstem infarction NOS |
|  | Xa00M | Wallenberg syndrome |
|  | Gyu63 | [X]Cerebrl infarctn due/unspcf occlusn or sten/cerebrl artrs |
|  | G6760 | Cereb infarct due cerebral venous thrombosis, non-pyogenic |
|  | X00DK | Posterior cerebral circulation haemorrhagic infarction |
|  | Gyu6G | [X]Cereb infarct due unsp occlus/stenos precerebr arteries |
|  | G63y1 | Cerebral infarction due to embolism of precerebral arteries |
|  | X00DJ | Anterior cerebral circulation haemorrhagic infarction |
|  | G63y0 | Cerebral infarct due to thrombosis of precerebral arteries |
|  | X00DB | Pure motor lacunar infarction |
|  | X00DD | Pure sensorimotor lacunar infarction |
|  | Xa00P | Weber syndrome |
|  | X00D4 | Infarction - precerebral |
|  |  |  |
| Stroke - unspecified | XaEGq | Stroke NOS |
|  | X00D1 | Cerebrovascular accident |
|  | G66.. | CVA - cerebrovascular accident (& unspecified [& stroke]) |
|  | XE2aB | Stroke and cerebrovascular accident unspecified |
|  | XE0X2 | (Cereb infarc)(cerebrovas acc)(undef stroke/CVA)(stroke NOS) |
|  | G667. | Left sided cerebral hemisphere cerebrovascular accident |
|  | X00DR | Stroke of uncertain pathology |
|  | G668. | Right sided cerebral hemisphere cerebrovascular accident |
|  | X00DT | Posterior circulation stroke of uncertain pathology |
|  | G664. | Cerebellar stroke syndrome |
|  | X00DE | Lacunar ataxic hemiparesis |
|  | X00DS | Anterior circulation stroke of uncertain pathology |
|  | G663. | Brainstem stroke syndrome |
|  | Xa00L | Benedict syndrome |
|  | X00DF | Dysarthria-clumsy hand syndrome |
|  | Xa1hE | Extension of cerebrovascular accident |
|  | XaQbM | Pure sensory lacunar syndrome |
